# Impaired Mitochondrial Bioenergetics under Pathological Conditions

**DOI:** 10.3390/life12020205

**Published:** 2022-01-29

**Authors:** Salvatore Nesci, Giorgio Lenaz

**Affiliations:** 1 Department of Veterinary Medical Sciences, Alma Mater Studiorum University of Bologna, Via Tolara di Sopra, 50, 40064 Ozzano Emilia, BO, Italy; 2 Department of Biomedical and Neuromotor Sciences, Alma Mater Studiorum University of Bologna, Via Massarenti 9, Pad 11, 40138 Bologna, BO, Italy

Mitochondria are the powerhouses of cells; however, mitochondrial dysfunction causes energy depletion and cell death in various diseases. The altered oxidative phosphorylation and ion homeostasis are associated with reactive oxygen species (ROS) production, resulting from the disassembly of respiratory supercomplexes and electron transfer chain disruption [1]. In pathological conditions, the dysregulation of mitochondrial homeostasis promotes Ca^2+^ overload in the matrix and ROS accumulation, which induce the mitochondrial permeability transition and pore formation responsible for morphological mitochondrial changes [2] linked to membrane dynamics, and ultimately, cell death.

The Special Issue, entitled “Impaired Mitochondrial Bioenergetics under Pathological Conditions”, includes 19 contributions, among which 5 are research articles and 14 are reviews. This research topic aimed to shed further light on the role of altered bioenergetics in diseases, a paramount argument in both basic and medical research. The energy transduction system has a supramolecular structure in order to better meet the bioenergetic demand of cellular activities.

Herein, the biochemical machinery alterations of oxidative phosphorylation (OXPHOS), hosted by the inner mitochondrial membrane, responsible for mitochondrial dysfunctions, and involved in several neurodegenerative and age-related diseases, have been discussed by considering: (*i*) the physio(patho)logical role of respiratory supercomplexes and (*ii*) the bifunctional features of ATP synthase as a life and death enzyme [3]. The structures of the mitochondrial electron transport chain in different supercomplexes (SCs) are all characterized by the presence of Complex III. Rugolo et al. highlight the genetic alterations that hinder the assembly of Complex III, which cause noticeable perturbation of the SCs architecture associated with several significant metabolic disturbances [4]. In mammalian mitochondria, Complex I is the largest respiratory complex, with 31 additional supernumerary subunits. Even if the accessory subunits are not necessary for enzyme activity, Zickermann’s group and Vik’s group discuss the mutations in these subunits which modify the Complex I assembly and lead to detrimental enzyme activity associated with many mitochondrial disease states [5,6]. A novel mutation of human mtDNA in the ATP6 gene has detrimental consequences on ATP synthase on yeast, with a pathogenicity resembling that which compromises human health [7]. However, altered expression of nuclear and mitochondrial genes of the ATP synthase is associated with neurodegenerative diseases and other age-related diseases in humans [8]. Indeed, Pamplona’s group report that ageing and the alteration of mitochondrial oxidative stress and lipid metabolism represent a positive loop in the progression of Alzheimer’s diseases [9]. Neurodegeneration and primary mitochondrial diseases, triggered by a defective respiratory chain, comprise impaired respiratory complex assembly and function. However, Needs et al. [10] consider that mitochondrial protein import is important for normal organelle physiology and observed an interlink with the regulation of respiratory complex assembly and function in human diseases. Moreover, in the paper by Franco et al., the human mitochondrial disorders that affect the OXPHOS activity have been suggested to be studied by using the *Saccharomyces cerevisiae* as a model to gain an understanding of the underlying molecular consequences of pathogenic mutations in mitochondria [11].

Ageing-related diseases are supported by mitochondrial-mediated regulated and programmed cell death in the myocardial structure, which promotes the development of heart failure [12]. Pinton’s group examines the disequilibrium in mitochondrial bioenergetics that leads to unbalance of the energy consumption/generation with increasing age and the imbalance in mitochondrial dynamics as impaired conditions in the failing heart [13]. Structural integrity and the coordination of mitochondrial enzyme activity in energy metabolism and redox homeostasis is also ensured by mitochondrial phospholipid cardiolipin (CL). However, diabetic cardiomyopathy and heart failure are cardiomyopathies described in Barth syndrome, a dysfunctional CL disease [14].

In metabolic diseases arising with age, mitochondrial ROS production might have a key role in inflammaging. Exercise induces improvements in mitochondrial function limiting ROS production by reducing inflammation and ensuring an adaptation of immune cell metabolism to fight against virus infection [15]. Indeed, Elesela and Lukacs consider that viruses exploit mitochondrial dynamics to induce negative impacts on the cell metabolism and promote viral life processes during infection [16]. The mitochondrial shaping and mitochondrial ROS production and release provide an intricate vicious circle, subject to maintaining physiological states or contributing to pathological conditions [17].

The accumulated evidence of cell bioenergetics of different tissues highlights a correlation with the pathogenesis of type 2 diabetes. In particular, mitochondrial dysfunction is related to the coupling mechanism of metabolism and the exocytosis of insulin and glucagon in diabete [18]. An increased risk of developing diabetes mellitus is found in patients with the m.3243A>G mutation, and excessive palmitate showed a negative effect on respiratory rates, promoting insulin resistance [19]. Therefore, measurement of mitochondrial bioenergetics by a new frozen respirometry protocol utilizing non-invasive analysis might facilitate the clinical monitoring of mitochondrial function in samples collected at remote sites [20]. In addition, mitochondrial oxidation and substrate oxidation measurement by in vitro myopathy assays was also considered a prognostic method for use on equine muscle biopsies [21].

In summary, studies on impaired mitochondrial bioenergetics in pathology could provide molecular tools to counteract diseases associated with mitochondrial dysfunctions.

## Data Availability

Not applicable.
